# Fermi function constrained deconvolution underestimates myocardial blood flow and myocardial perfusion reserve regardless of saturation correction of arterial input curve

**DOI:** 10.1186/1532-429X-18-S1-P87

**Published:** 2016-01-27

**Authors:** Akimasa Yamada, Masaki Ishida, Takashi Ichihara, Takahiro Natsume, Shinsuke Tsuge, Yoshitaka Goto, Mio Uno, Motonori Nagata, Yasutaka Ichikawa, Kakuya Kitagawa, Hajime Sakuma

**Affiliations:** 1 grid.412075.5Radiology, Mie University Hospital, Tsu, Japan; 2 grid.256115.4School of Health Science, Fujita Health University, Toyoake, Japan

## Background

Myocardial blood flow (MBF) can be quantified from arterial input function (AIF) and myocardial output function in perfusion MRI by using tracer kinetic modeling. Fermi function constrained deconvolution has been widely used to determine MBF and myocardial perfusion reserve (MPR). However, stress MBF and MPR in healthy subjects quantified by the Fermi deconvolution approach were reported to be 2 to 3 mL/min/g and 2 to 3, respectively, which are substantially smaller than those quantified by ^15^O-water PET (3 to 5 mL/min/g and 3 to 5, respectively). Tissue compartment model analysis with Patlak plot is an alternative approach to estimate MBF and MPR. In a recent study employing corrections for AIF saturation and extraction fraction of gadolinium contrast medium, MBF and MPR by model-based Patlak plot method showed an excellent agreement with those quantified by ^15^O-water PET. In the current study, we compare MBF and MPR calculated by Fermi function constrained deconvolution with those by model-based Patlak plot method.

## Methods

Nine subjects (6 men, 65.1 ± 6.8 years) with normal coronary arteries were evaluated with a 3.0T MR system. First-pass perfusion MR images were obtained with a saturation recovery TFE sequence every heart beats. In order to perform saturation correction of the blood signal, we initially obtained first-pass MR images by administrating 10x diluted Gd-DOTA (0.003 mmol/kg). Then first-pass MR images were acquired at rest and during ATP stress with a Gd-DOTA dose of 0.03 mmol/kg. MBF was quantified by a Fermi constrained deconvolution with and without AIF saturation correction using an AHA 16 segment model. MBF was also calculated by model-based Patlak plot method with the corrections of AIF saturation and extraction fraction of gadolinium contrast medium.

## Results

**Figure 1:**
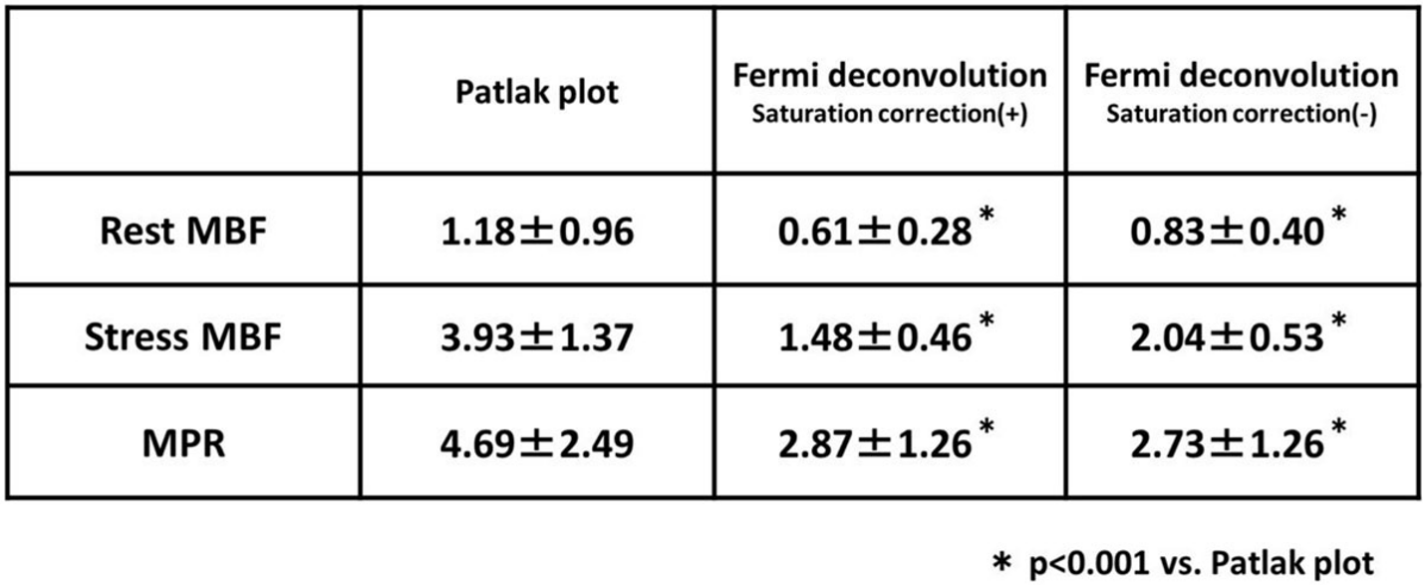
**MBF and MPR by Patlak plot and Fermi function deconvolution**.

Figure 1 summarizes the MBF and MPR calculated by model-based Patlak plot method and Fermi function constrained deconvolution. Rest MBF, stress MBF, MPR by Patlak plot method were 1.18 ± 0.96 mL/min/g, 3.93 ± 1.37 mL/min/g and 4.69 ± 2.49,respectively, which are comparable to those quantified by^15^O-water PET in previous reports. The MPR calculated by Fermi function constrained deconvolution, either with (2.87 ± 1.26, p < 0.001) and without (2.73 ± 1.26, p < 0.001) saturation correction, was significantly smaller than the MPR (4.69 ± 2.49) obtained by model-based Patlak plot method. The use of Fermi function constrained deconvolution is associated with underestimation of the MBF particularly during stress, which cannot be compensated by correcting AIF saturation (stress MBF of 1.48 ± 0.46 mL/min/g with saturation correction, 2.04 ± 0.53 mL/min/g without saturation correction).

## Conclusions

The current results demonstrated that Fermi function constrained deconvolution underestimates MPR regardless of saturation correction of arterial input function.

